# Supplementary material for: Xylo-Oligosaccharides in Prevention of Hepatic Steatosis and Adipose Tissue Inflammation: Associating Taxonomic and Metabolomic Patterns in Fecal Microbiomes with Biclustering
Source: Int J Environ Res Public Health. 2021 Apr 12;18(8):4049. doi: 10.3390/ijerph18084049 (PMC8068902; doi:10.3390/ijerph18084049)
Supplement: Supplementary file 1 [file ijerph-18-04049-s001.pdf]

Table 1. Average body and liver weights in each diet group. Effects of XOS and fat tested with UNIANOVA in IBM SPSS.

	HFD	HFD + XOS	LFD	LFD + XOS	Fat effect	XOS effect	Interactive effect
**Body weight (g)**	510 ± 36	508 ± 29	414 ± 20	433 ± 26	[F (1, 74775) = 92.1, p<0.001]	NS	NS
**Liver weight (g)**	13.64 ± 1.14	13.48 ± 0.62	12.82 ± 0.82	12.83 ± 0.80	[F (1, 5.009) = 6.7, p=0.014]	NS	NS
**Liver/body weight ratio**	0.0277 ± 0.0021	0.0268 ± 0.0014	0.0311 ± 0.0019	0.0302 ± 0.0013	[F (1, 0.000) = 47.5, p<0.001]	NS	NS

Table S2. The prediction performance of the classifier model for all diet groups (HFD, HFD + XOS, LFD, LFD + XOS), dietary fat (high, low) and XOS ingestion (true, false). Accuracy and f1 score for each run derived from 5-fold cross validation.

**Average model accuracy-% and F1 scores from cross-validation. XGBoost classifier**			
		**Only metabolites**	
	**Diet group**	**Fat content**	**XOS supplementation**
**all biclusters**	42.5 +/- 12.7, 0.43	**92.5 +/- 10, 0.92**	**60 +/- 9.4, 0.55**
**SCFA bicluster**	50 +/- 11.2, 0.5	**92.5 +/- 10, 0.92**	57.5 +/- 15, 0.58
**Product bicluster**	32.5 +/- 15, 0.33	77.5 +/- 9.4, 0.78	35 +/- 14.6, 0.26
**TMA bicluster**	27.5 +/- 14.6, 0.28	60 +/- 14.6, 0.61	47.5 +/- 14.6, 0.48
**AA bicluster**	47.5 +/- 9.4, 0.48	75 +/- 7.9, 0.74	**62.5 +/- 7.9, 0.62**
**Isovalerate bicluster**	37.5 +/- 7.9, 0.38	**85 +/- 14.6, 0.85**	52.5 +/- 18.4, 0.49
		**Only genera**	
	**Diet group**	**Fat content**	**XOS supplementation**
**all biclusters**	50 +/- 17.7, 0.5	**82.5 +/- 10, 0.83**	42.5 +/- 12.7, 0.45
**SCFA bicluster**	45 +/- 21.8, 0.45	**87.5 +/- 0, 0.87**	40 +/- 18.4, 0.35
**Product bicluster**	45 +/- 12.7, 0.45	**82.5 +/- 10, 0.79**	40 +/- 9.4, 0.35
**TMA bicluster**	37.5 +/- 13.7, 0.38	60 +/- 16.6, 0.58	**60 +/- 16.6, 0.57**
**AA bicluster**	30 +/- 17, 0.3	75 +/- 13.7, 0.63	45 +/- 15, 0.38
**Isovalerate bicluster**	37.5 +/- 13.7, 0.38	**92.5 +/- 6.1, 0.9**	40 +/- 5, 0.39
